# Lactation and Neonatal Nutrition: Defining and Refining the Critical Questions

**DOI:** 10.1007/s10911-012-9261-5

**Published:** 2012-07-01

**Authors:** Margaret C. Neville, Steven M. Anderson, James L. McManaman, Thomas M. Badger, Maya Bunik, Nikhat Contractor, Tessa Crume, Dana Dabelea, Sharon M. Donovan, Nicole Forman, Daniel N. Frank, Jacob E. Friedman, J. Bruce German, Armond Goldman, Darryl Hadsell, Michael Hambidge, Katie Hinde, Nelson D. Horseman, Russell C. Hovey, Edward Janoff, Nancy F. Krebs, Carlito B. Lebrilla, Danielle G. Lemay, Paul S. MacLean, Paula Meier, Ardythe L. Morrow, Josef Neu, Laurie A. Nommsen-Rivers, Daniel J. Raiten, Monique Rijnkels, Victoria Seewaldt, Barry D. Shur, Joshua VanHouten, Peter Williamson

**Affiliations:** 1 University of Colorado School of Medicine, Aurora, CO 80045 USA; 2 University of Colorado School of Public Health, Aurora, CO 80045 USA; 3 Arkansas Children’s Nutrition Center, University of Arkansas for Medical Sciences, Little Rock, AR 72002 USA; 4 University of Illinois, Urbana, IL 61801 USA; 5 University of California Davis, Davis, CA 95616 USA; 6 Baylor College of Medicine, Children’s Nutrition Research Center, Houston, TX 77030 USA; 7 Human Evolutionary Biology, Harvard University, Cambridge, MA USA; 8 University of Cincinnati School of Medicine, Cincinnati, OH 45229 USA; 9 Rush University Medical Center, Chicago, IL 60612 USA; 10 University of Florida, Gainesville, FL 32611 USA; 11 Duke University, Durham, NC 27710 USA; 12 Yale University, New Haven, CT 06520 USA; 13 The University of Texas Medical Branch, Galveston, TX 77555 USA; 14 Pfizer Nutrition, Madison, NJ 07940 USA; 15 Faculty of Veterinary Science, University of Sydney, NSW 2006 Australia; 16 University of Colorado Denver, Graduate School, Aurora, CO 80045 USA; 17 Eunice Kennedy Shriver National Institute of Child Health and Human Development (NICHD), National Institutes of Health (NIH), Bethesda, MD 20892 USA; 18 Department of Obstetrics and Gynecology, Reproductive Sciences/Mail Stop 8613, 12700 E. 19th Ave, Room 3400D, Aurora, CO 80045 USA

**Keywords:** Lactation, Infant nutrition, Human milk, Breastfeeding, Mammary gland development, Human nutrition, Preterm birth, Obesity, Undernutrition, Lactational programming, Milk

## Abstract

This paper resulted from a conference entitled “Lactation and Milk: Defining and refining the critical questions” held at the University of Colorado School of Medicine from January 18–20, 2012. The mission of the conference was to identify unresolved questions and set future goals for research into human milk composition, mammary development and lactation. We first outline the unanswered questions regarding the composition of human milk (**Section I**) and the mechanisms by which milk components affect neonatal development, growth and health and recommend models for future research. Emerging questions about how milk components affect cognitive development and behavioral phenotype of the offspring are presented in **Section II**. In **Section III** we outline the important unanswered questions about regulation of mammary gland development, the heritability of defects, the effects of maternal nutrition, disease, metabolic status, and therapeutic drugs upon the subsequent lactation. Questions surrounding breastfeeding practice are also highlighted. In **Section IV** we describe the specific nutritional challenges faced by three different populations, namely preterm infants, infants born to obese mothers who may or may not have gestational diabetes, and infants born to undernourished mothers. The recognition that multidisciplinary training is critical to advancing the field led us to formulate specific training recommendations in **Section V**. Our recommendations for research emphasis are summarized in **Section VI.** In sum, we present a roadmap for multidisciplinary research into all aspects of human lactation, milk and its role in infant nutrition for the next decade and beyond.

## Introduction and Impact

“Mothers and infants form a biological and social unit; they also share problems of malnutrition and ill-health” [WHO, Infant and Young Child Nutrition: Global Strategy on infant and young child feeding. 2012]

From an evolutionary, nutritional and economic standpoint, human milk is the ideal food for the human infant for the first months of life. Exclusive breastfeeding for the first 6 months followed by breastfeeding supplemented with appropriate complementary foods for 1 year or longer continues to be the recommendation of the American Academy of Pediatrics (AAP) [1], Centers for Disease Control and Prevention (CDC) [2] and the World Health Organization [3]. In the US these recommendations are currently met by only 13 % of mothers leading to multiple initiatives by these agencies to improve the rates of breastfeeding initiation, duration and exclusivity. An important strategy is advocacy for “baby friendly hospitals” [3] to improve breastfeeding practices in US hospitals and communities. However, it is possible that a focus on specific populations for which feeding human milk makes a critical difference would have the most impact.

Milk is a complex fluid that exerts effects far beyond its nutritional value. Yet even its composition is not well-defined. A full appreciation of the range of nutritive and non-nutritive components of milk and their secretion could be translated into actionable policies that could impact the initiation, duration and exclusivity of breastfeeding as well as contribute to the intelligent design of human milk substitutes.

**Fig. 1:**
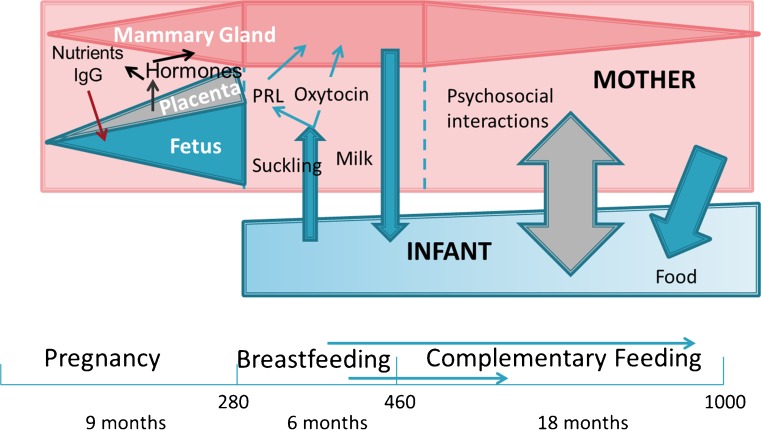
The first 1,000 days. The crucial periods in infant metabolic and cognitive development are thought to occur during the first 1,000 days of fetal/infant life and can be divided into three phases: pregnancy, breast (or formula) feeding, and a period of increasing complementary feeding. General maternal/fetal and maternal/infant interactions are indicated for each phase

Even prior to conception, the health and nutritional status of the mother have a profound influence on fetal development and that of the mammary gland [4, 5]. The timing of birth [6], obesity [7], maternal undernutrition [8] and other maternal health problems [4, 9] are major factors that influence the nutritional needs and health of the neonate. For these reasons the role of human milk in infant nutrition must be studied in light of the changing dynamic that exists a between mother and her child from before conception through pregnancy and into postnatal life (Fig. 1). In addition, a clear understanding of the factors that affect the ability of the mother to produce sufficient milk of appropriate composition for her infant is critical in developing evidence-based programs/policies to support breastfeeding.

### Economic Impact

The financial impact of improving neonatal nutrition in at-risk populations is enormous. One major impact pertains to the consequences of preterm birth. It is estimated that 65,000 very low birth weight infants (birth weight <1,500 g) are born in the United States each year. If the average hospital cost for mother and infant is ~$140,000 (RW Hall, personal communication), the total annual cost in the US is more than $9 billion. If feeding human milk or its important components reduced the incidence of necrotizing enterocolitis (NEC) and other serious medical problems by even 15 %, savings of over $1 billion per year could be realized. A similar economic impact could be realized if the obesity epidemic were reduced. The economic burden of this epidemic is considered to have four elements, namely, increased costs of medical care, costs of lost productivity, transportation costs, and human capital accumulation [10] estimated together to cost at least $250 billion per year. If breastfeeding reduced the incidence of obesity in susceptible individuals by even 10 % then savings of up to $25 billion could be realized. A third area of high impact is that of malnutrition on a global scale. Malnutrition affects 900 million children and their mothers, with an inestimable economic impact. If the balance of feeding human milk and complementary foods were optimized to minimize growth stunting in the first two years in these individuals, the impact would be enormous [8].

### Social Impact

Despite national and international initiatives on breastfeeding, we lack an in depth understanding of both the biological and psychosocial aspects of breastfeeding. Studies of the mechanisms by which milk components affect all aspects of infant development are in their infancy, but it is increasingly clear that at-risk infants have special nutritional needs and that, for such infants, the current uniform breastfeeding recommendations are sub-optimal. These deficits seriously impact the information available to health care providers and families alike. Such knowledge could be translated into science-based consumer information and professional “toolkits” to help more women successfully meet their own breastfeeding goals and achieve the best nutrition for their infants.

In this paper we outline the specific questions that arose at this wide-ranging conference as being important for research in the next decade. These questions focus on the composition of milk, the effects of milk components on neonatal development, the regulation of mammary development and milk secretion, and finally problems specific to at-risk populations.

## Summary of Workshop Proceedings

Topic I Human Milk Components and their Function
  - What is the composition of human milk?
  - What are the detailed mechanism by which milk components promote neonatal development?
  - What is the role of human milk in establishing the infant microbiome?
  - How do environmental agents and pharmaceuticals in milk affect infant development?
  - What is the role of the dairy products in infant nutrition?
Topic II Effects of milk on behavioral and cognitive development
  - What are the components of milk that affect cognitive development and how do they work?
  - What elements of breastfeeding affect behavioral phenotype?
  - Methods for studying the effects of milk on cognitive and behavioral development.
Topic III Maternal factors affecting milk volume and composition.
  - How do hormones and metabolism coordinately program the mammary gland for lactation?
  - What are the genetic and epigenetic elements that affect heritability of mammary gland development and lactation-related traits?
  - What are the mechanisms by which maternal nutrition, disease, and metabolic status affect milk composition and volume?
  - What breastfeeding practices prevent inadequate lactation performance?
  - How do environmental agents and pharmaceuticals affect milk volume and composition?
Topic IV Breastfeeding and the at-risk neonate
  - What is the role of human milk in the nutrition of the preterm infant?
  - What is the role of breastfeeding in preventing obesity in susceptible populations?
  - What is the role of human milk in preventing growth stunting?
  - A community approach to lactation studies in at-risk human populations.
Topic V Training the future lactation biologist
Topic VI Imperatives for advancing the field

## Topic I: Human Milk Components and Their Function

Defining the composition of milk and the specific individual and combined effects of its components on infant outcomes is fundamental to optimizing the survival, health, and development of the neonate in clinical and other settings. Human milk is complex [11] and has a host of non-nutritive functions including protection against infection, reducing inflammation [12], promoting intestinal, immune and cognitive development, and stimulating establishment of the unique gut microbiome of the breastfed infant [13, 14]. In this section we summarize what is known about human milk composition and its variation, where we are with our knowledge of the function of milk components, the role of the microbiome in infant development, and the potential effects of drugs and toxins on the infant.

- What is the composition of human milk?
  (Contributors: German, Lemay, Lebrilla)

- **Research Target: A complete analysis of the structure and concentration of human milk components as well as factors influencing their variability between women and throughout lactation.**

New analytical tools that provide more quantitative and sensitive methods have become available over the last decade [15–17] and are revolutionizing our understanding of the diversity of molecules in milk. These tools can now be used to provide a catalogue of the structure of all proteins, oligosaccharides, glycoconjugates, metabolites, lipids and polynucleotides (RNA, DNA) in the various fractions of milk.

### Methods

Advantage should be taken of large epidemiological studies to bank samples of human milk at defined intervals postpartum. Analyzing cellular mRNA present in the cytoplasmic crescents of the milk fat globule provides a window into the transcriptome of the mammary alveolar cell in lactating women [18] that can be used to dissect the effects of maternal states such as obesity and stress on mammary cell function. For this reason particular importance should be given to proper sampling and storage of the lipid fraction of milk. It is also essential that the collection and storage of these milk samples be accompanied by systematically recorded detailed information regarding the health, nutrition, immune function and reproductive state of the woman providing the sample as well as the growth and development of her infant.

b. What are the detailed mechanisms by which milk components promote neonatal development? (Contributors: Donovan, Contractor, Goldman, Meier, Forman)

- **Research Target: A detailed knowledge of the functional mechanisms by which milk components acting both individually and together, promote intestinal maturation, protect against infection and inflammation, and stimulate immune development in the infant.**

In general, the variations in milk composition as well as the mechanism of action of many bioactive components of human milk in the infant remain poorly characterized although these factors contribute significantly to infant development [12]. Where certain constituents are low or lacking in the milk of particular mothers, opportunities for supplementation may exist. For infants whose mothers are unable to breastfeed, a greater understanding of bioactive factors may help improve the development of human milk substitutes in relation to infant health. Thus a fuller appreciation of the range of function of the nutritive and non-nutritive components of human milk has significant potential to translate to improved public health.

Most studies have focused on comparing human milk and formula. However, by 6 months of age, most infants are receiving both maternal milk and infant formula. How this combined feeding strategy impacts resistance of the infant to infection and other aspects of development is virtually unknown. As studies are developed they should be designed to include interactions between components of human milk and complementary foods, including formula.

### Intestinal Development

The gastrointestinal (GI) tract undergoes significant postnatal growth and functional adaptation in response to feeding (reviewed in [12, 19]). The transition from predominantly parenteral support via the umbilical cord to enteral support via human milk or formula presents a challenge to the researcher who must come to understand the interaction between GI mucosal surfaces and environmental stimuli such as dietary antigens, commensal and pathogenic microbiota and their products [20, 21]. In addition, hormones and growth factors constitute a class of bioactive proteins and peptides in milk [19], the best-studied of which include insulin-like growth factor-1 (IGF-1), epidermal growth factor (EGF), and transforming growth factor (TGF)-β. Our current knowledge of the mechanisms by which these and other bioactive components act in the neonatal intestinal has been limited by the complexity of human milk, an inadequte understanding of the normal progression of postnatal intestinal development and its underlying regulation, and the limited number of non-invasive methods available to assess the impact of milk components. Recently, Chapkin and colleagues used exfoliated epithelial cells to assess intestinal gene expression, documenting differences between cells from human milk- and formula-fed infants [22]. This non-invasive research model along with studies in neonatal pigs [23] and, potentially, monkeys [24] provides an important system to understand the effects of milk components on GI development.

### A Note About Colostrum

Colostrum is secreted by lactating women in small quantities (~100 ml per day) for the first 2 days after birth, during which its composition is constantly changing [25]. In species that, unlike the human, do not transfer systemic immune components across the placenta, the IgG in colostrum provides systemic immunity to the offspring while their endogenous immune systems develop [26]. Human colostrum has very little IgG and does not directly provide systemic immunity, but does have high concentrations of protective components such as secretory immunoglobulin A (sIgA), lactoferrin and human milk oligosaccharides (HMO) [27], which provide protection to mucosal surfaces. Human colostrum also contains growth factors and cytokines such as transforming growth factor (TGF)-β, interleukin-(IL)-10, and erythropoietin that can suppress inflammatory responses in the immature neonatal intestine [12]. Detailed knowledge of the time course of secretion of these colostrum components through lactation as well as the mechanisms by which they act to promote intestinal development is likely to lead to optimized protocols for feeding human milk and/or its components to at-risk infants (See Topic IV).

### Protection Against Infection

Because conferring resistance to infection is a major function of human milk and is critically important in developing countries, it is imperative to understand which components of milk are most important for this effect and their mechanisms of action. Many milk proteins such as lactoferrin and lysozyme have direct anti-microbial effects [28–30]. Others such as oligosaccharides may act as decoy receptors to block adhesion of pathogens to epithelial cell surfaces [31]. Human milk nucleotides have been shown to stimulate an increased host immune response [32, 33].

An improved understanding of antimicrobial components in human milk may foster the development of resources to improve the antimicrobial quality of animal milks. For example, elevated lysozyme concentrations in the milk of transgenic goats improved duodenal development and reduced the levels of inflammation and *E.coli* in the ileum of neonatal pigs [34]. This experiment illustrates both the potential of transgene-expressed protective substances in animal milks and an elegant use of the piglet to study the function of milk components.

### Immune Development and Altered Allergic Responses

Systemic and mucosal immunity begin to develop in utero, and continue to develop from the postnatal period through early childhood. The neonatal mucosal immune system is relatively devoid of IgA producing B cells. Secretory IgA available through human milk may serve a compensatory role as the infant’s IgA producing B cells develop and differentiate following exposure to antigens ex-utero. Cues from the microenvironment including the gut microbiome [35] and nutritional components regulate the development of the immune response both locally in the intestine and systemically. An important example is the observed role of vitamin A metabolites acting via the retinoic acid receptor to suppress development of Th17 cells [36]. Other molecules in milk that have receptors expressed by cells of the immune system include lactoferrin (lactoferrin receptor) [37] and acidic sialic acid-containing carbohydrates that may potentially bind to sialic acid binding receptors, also known as SIGLECs [38]. Data from animal models suggest that allergen exposure via human milk mediates development of regulatory T cells in neonates that, in turn, protect the animal from developing asthma when later challenged with the allergen [39]. Whether these findings translate to humans remains an important question given that the incidence of childhood allergy continues to increase [40].

Studies of the immunological properties of human milk and its effects upon the infant should provide important insights into several issues:

1. Regulation of the development of the infant’s immune system and inflammatory responses,
2. The optimal duration of breastfeeding
3. The role of milk in the future programming of diseases mediated by infections, immunologic events or inflammation.

Understanding of these issues would afford insight into novel means to impact human health and development.

c. What is the role of human milk in establishing the infant microbiome? (Contributors: Frank, Janoff)

**Fig. 2:**
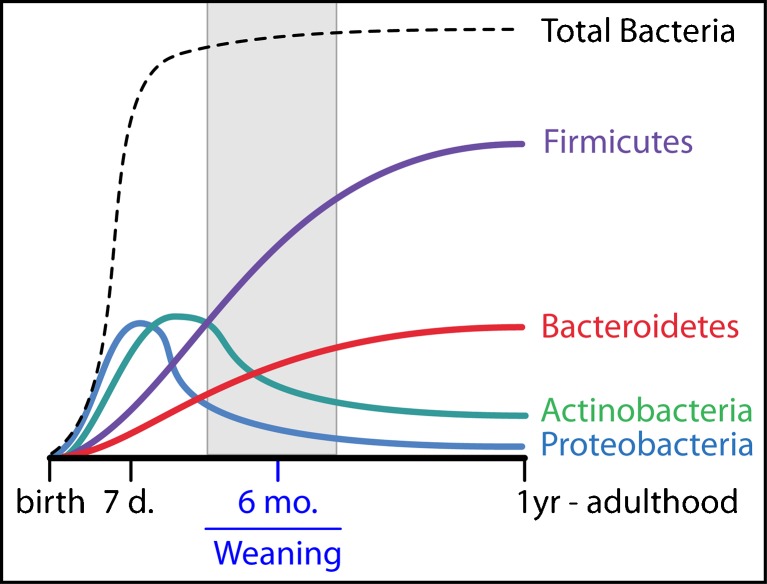
Development of the microbiome in the human infant. Figure modified from references 41 and 42

The infant GI tract is immunologically and microbiologically naive at birth. The early days and weeks following birth mark a period of profound change in the neonatal GI tract as the number of bacteria increase from near-sterility to levels found in adults [41, 42] (Fig. 2). Over the ensuing 12–24 months, the GI microbiota transitions to domination by obligate anaerobes of the phyla *Bacteroidetes* and *Firmicutes*. In parallel with this microbial succession, the gut-associated lymphoid tissue (GALT) and other components of the innate and adaptive mucosal immune system reach maturity [13]. In mice the presence of microbial communities in the gut is an essential driver of GALT development [43, 44]. Analogously, the human GI microbiome likely is a critical determinant of infant immune function.

Both diet (breastfeeding vs. formula feeding) and mode of delivery of the infant (vaginal vs. Caesarean) are significant early determinants of the kinds and quantities of microbes that colonize the neonatal intestines [45]. For example, Penders et. al. demonstrated that at 1 month of age, the potentially “beneficial” *Bifidobacteria* strain was more prevalent and present in higher numbers among 700 exclusively breastfed Dutch infants than in 232 formula-fed infants and 98 infants who received mixed feeding [46]. In contrast, microorganisms such as *Clostridium difficile, Bacteroides fragilis* and *E. coli* that are believed to have a higher inflammatory potential were present in higher numbers in the latter group, as were *Lactobacilli*. Human milk oligosaccharides are fermentable and selectively nourish the growth of specific strains of *Bifidobacteria* found among the microflora of the breastfed infant [14, 47]. Data from in vitro studies suggest that proteolytic fragments of α-lactalbumin, lactoferrin, and the secretory component of the polyimmunoglobulin receptor (35) can selectively stimulate the growth of *Bifidobacteria* [48]. An important unknown is the nature of other digestion products of milk proteins that may play a role in specifying the infant microbiome.

Despite the great progress made recently in linking the GI microbiome with nutrition, feeding practices and immunity, many important questions remain unanswered:

1. How do the microbes that colonize the developing gut program immune development?
2. Do perturbations of the GI microbiome resulting from mode of feeding, delivery, or antibiotic exposure have long term health consequences?
3. Can a dysfunctional neonatal microbiome be bioremediated by dietary interventions?

Recently it has become clear that a complex microbiome exists in human milk [49] although whether these microflora contribute to the infant microbiome is not clear. Further, the source of these microorganisms and the factors that influence their presence are completely unknown.

d. How do environmental agents and pharmaceuticals in milk affect infant development? (Contributors: Badger, Meier)

- **Research Target: Compile a complete database of commonly used therapeutic drugs that includes their rate of secretion into milk as well as effects on infant health and development.**

In 2006 more than 1 million mothers in the US cited the need to take a wide variety of medications as the reason they elected not to breastfeed [50]. In addition mothers are unfortunately not immune to the effects of recreational drugs and cigarette smoking. Recent articles indicate that codeine and the nicotine derived from cigarette smoking [51] are secreted into the milk with significant effects on the infant. These effects, which can result in infant death [52], emphasize the potential dangers of maternal drug use for the infant. Likewise, beyond the established pronounced effects of alcohol consumption on the unborn fetus, alcohol intake by lactating mothers alters the sensory properties of human milk and infant nursing behavior [53].

Many mothers have conditions that legitimately require consistent drug therapy including psychiatric problems, heart disease, hypertension and rheumatic illnesses. For rheumatic diseases good recommendations about drugs that can and cannot be used during pregnancy and lactation are available [54]. There is considerable information about levels of antidepressants in maternal plasma and human milk, but very little information exists about their effects on infant behavioral outcomes [55]. Caregivers and potential breastfeeding mothers must have appropriate information regarding the effects of specific drugs on breast milk and on infant health in order to make appropriate decisions about breastfeeding.

e. What is the role of dairy products in infant nutrition? (Contributors: Williamson, German, Hovey)

The dairy industry currently embraces an emerging potential for personalized nutrition and designer milks. These concepts are intertwined. In the context of infant formula, they translate to a design that is (1) humanized, (2) specialized for clinical use, (3) supplemented for optimized neonatal health.

Important questions for future consideration are:

1. To what extent can the functions of human milk components be mimicked by the corresponding molecules in milk from other species?
2. Can specific components of human milk be replaced by non-homologous components?
3. Can molecules synthesized in yeast, fungi or plants, or recombinant molecules, be used to supplement formula with positive outcomes?

A good deal of current attention has focused on oligosaccharides as likely-important substrates for gut biota [14]. These molecules have been associated with resistance to allergies and infections as they are thought to foster a robust immune system. An important question for future research is to what extent bovine milk oligosaccharides can support “healthy” infant-type growth of *Bifidobacteria*. The need for further research into the detailed composition of cow and goat milks is important given that its synthesis and post-harvest processing can provide for new, possibly designer molecules that are able to enhance human health.

## Topic II. Effects of Milk on Cognitive and Behavioral Development

- **Research Target: Determine the effect of specific and interacting components of milk on brain development and the behavioral phenotype.**

An important but poorly understood area is the effect of mother’s-own milk on both cognitive development and the behavioral phenotype. Optimizing cognitive development is critical given that children with impaired cognition adapt less well to stressful events, are more vulnerable to anxiety and attention deficits and often require expensive special education programs. In many parts of the world, including the US, cognitive impairment contributes to the cycle of poverty and disease [56, 57]. Moreover, cognitive dysfunction is a major co-morbidity in a number of neuropsychiatric diseases that manifest later in life [58]. Understanding the role of milk components in cognitive and behavioral development is therefore a crucial area for research.

- What are the components of milk that affect cognitive development and how do they work? (Contributors: Hinde, Donovan)

Human milk has relatively high concentrations of molecules that foster brain development including choline, sialic acid and long-chain polyunsaturated fatty acids (LC-PUFA) [59]. Prenatal exposure to choline and its long-term effects on hippocampal development, learning, memory and emotional behavior have been studied extensively in animal models [60], although relevant data for humans are lacking. More recently, attention has been focused on sialic acid (SA) given that accretion of SA-containing gangliosides in brain increases nearly 3-fold from week 10 of gestation through 5 years of age [61]. Likewise, an important role for LC-PUFA in brain maturation has long been suspected; these lipids, particularly docosahexaenoic acid (DHA) and arachidonic acid (AA), are present in human milk at much higher concentrations than in bovine milk and can be altered by diets containing large amounts of fish oil [59]. Although infant formula is often supplemented with LC-PUFA [62], any long term effects of these components on cognitive development have been difficult to document in term infants [63].

Important studies by Caspi and colleagues showed that the effects of breastfeeding on IQ can be mediated by genetic variation in *FADS-2,* a gene involved in desaturating LC-PUFA [64]. Compared to infants with no mutation, feeding human milk in infants who were homozygous for this mutation increased their IQ at 6.5 years of age [65]. Another group found that mothers homozygous for the mutation did not increase the proportion of LC-PUFA in their milk with increasing fish and fish-oil intake [66]. The implications of these findings for supplementation of mothers and/or infants with LC-PUFA have yet to be established. Importantly, however, these data clearly illustrate two major points.

1. Gene-diet interactions exist that may affect both milk composition and infant development.
2. Any effects of genetic polymorphisms on infant outcomes may be masked when only mean values are compared across populations. The ends of the bell-shaped curve may give important information about the contribution of specific gene-diet combinations.

b. What elements of breastfeeding affect behavioral phenotype? (Contributors: Hinde, Friedman)

Behavioral phenotype is defined as the observable “behavioral characteristics of an individual resulting from the interaction of its genotype with the environment, often manifested in a suite of co-varying behaviors” [67]. Feeding mother’s-own milk reflects a complex physiological and behavioral negotiation between the mother and the infant that begins during pregnancy [68] when the mammary gland develops functionally and is potentially sensitive to fetal signals [69]. A number of components in milk subsequently impart specific effects on development. For example, relaxin in sow’s milk in the early post-partum period programs uterine development [70] giving rise to what Bagnell termed the “lactocrine hypothesis”. Behavioral interactions between mothers and their infants during lactation can influence milk removal and thereby alter milk synthesis [69]. But are there lactocrine influences on the infant’s behavioral trajectory?

Studies in the rhesus macaque strongly suggest that that the answer to this question is “Yes”. Specifically, the cortisol content of the mother’s milk was significantly related to infant’s behavioral phenotype [71]. Further, Hinde and Capitanio [71] demonstrated that milk energy density and yield predict behavioral outcomes for both male and female infants. Importantly, the observed temperament and behavioral outcomes reflected available milk energy from months earlier, not at the time of assessment, suggesting that early nutrition organizes, or programs, infant behavior during critical windows of development. In another setting a maternal diet high in fat (HFD) perturbed the central serotonergic system of offspring in monkeys leading female offspring from HFD-fed mothers to exhibit increased anxiety in response to threatening novel objects. These findings have important clinical implications as they demonstrate that a maternal HFD during gestation and lactation, independent of obesity, may increase the risk of developing behavioral disorders, such as anxiety, in the offspring [72].

A recent report from the Cambridge Baby Growth Study suggested that breast- and mixed-fed human infants had more labile temperaments than formula-fed infants [73]. Is this a lactocrine effect of milk cortisol? The relationship between maternal cortisol and infant temperament was examined in breast- and formula-fed infants [74]. At 2 months postpartum breastfeeding mothers with higher plasma cortisol (used as a proxy for milk cortisol) rated their infants significantly more fearful than did breastfeeding mothers with lower plasma cortisol concentrations. Importantly, this relation was absent when infants were formula-fed. These data support the hypothesis that the cortisol ingested via milk has a lactocrine effect on infant temperament.

c. Methods for studying the effects of milk on cognitive and behavioral development (Contributors: Badger, Donovan, Hinde, Friedman,)

- **Research Target: Understand the effects of mother’s own milk and its components on the trajectory of infant cognitive and behavioral development.**

**Fig. 3:**
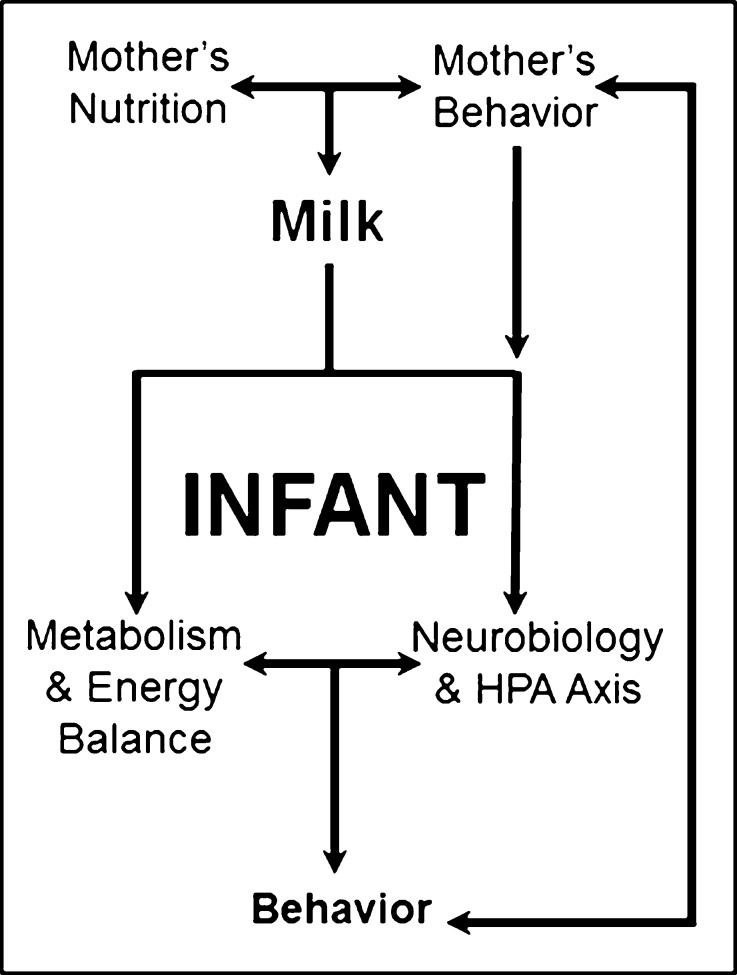
Interactions of components influencing the behavioral phenotype. Adapted from reference 68

Studies in humans and animal models will be needed to unlock the role that specific molecules and maternal behaviors play in early development in the neonate. When investigating potential lactocrine influences on infant behavioral development a number of interacting factors must be considered as shown in Fig. 3. Focused attention needs to be placed on subjects whose developmental trajectories deviate significantly from the norm. In such a population it is often possible to identify specific, genetic or environmental factors that impact infant development. Examining the sources of variation (environmental, psychological, physiological, metabolic, dietary, lifestyle, social) and the consequences of that variation for the infant (immune, neurobiological, physical, and behavioral growth/development) will allow us to promote better breastfeeding practices while improving formulas for vulnerable populations.

### Studies in Human Mother-Infant Pairs

Specific testing of brain function, especially within the first year of life, has provided mixed, albeit variable, results with respect to a comparison between breastfeeding and formula. An elegant technique known as event-related potentials (ERP) can be used to measure infant responses to stimuli that are potential important in language acquisition. However, experiments using this technique have yielded mixed results with respect to differences between formula- and breastfed infants [75–77]. The caveat is that all of these ERP studies were conducted at a single age; infants develop rapidly in the first few months of life and acquire processing abilities at slightly different ages, so these differences could reflect the individual developmental trajectories. None-the-less these studies indicate that sophisticated cognitive tests are available to test hypotheses about behavior-modifying effects of specific milk components. It is important to take into account factors such as parent education, IQ, socio-economic status, maternal-infant interactions, and home environment if these studies are to provide reliable information about the effects of milk components.

### Studies in Non-Human Primates

Compared to the altricial young of rodents, primate infants begin to engage behaviorally with a wide social network including parents, siblings, friends, and other conspecifics soon after birth. Infants depend on mother’s own milk, or an alternative, not only for nutrition but to sustain these social interactions during the post-natal period. Careful studies in primate populations relating variations in milk composition and volume among individual mothers to their nutrition, health, body mass, social condition may identify lasting consequences for neurodevelopment and behavior. Importantly, among primates the early expansion of an primate infant’s behavioral repertoire is co-incident with critical windows of neurodevelopment [78].

A better understanding of the neurobiological underpinnings of behavioral outcomes is needed to extend these findings for translation to human health. Although non-human primates are likely the most similar to humans for investigating how milk influences neuro-psycho-biological development and social behavior, additional animal models particularly pigs and rodents will provide crucial, complementary information. The dairy science literature may also be a source of important information given that milk production is closely linked to the behavior of the cow during milking [79].

## Topic III: Maternal Factors Affecting Milk Volume and Composition

Hormones coordinate mammary gland development and lactation with reproductive development and needs of the infant (Fig. 1) [80]. During early pregnancy, for example, ovarian progesterone and pituitary prolactin initiate and promote alveolar proliferation. As pregnancy progresses placental hormones play significant but poorly understood roles in promoting alveolar differentiation. The abrupt fall in progesterone at parturition fosters secretory activation which depends on prolactin and, as recently elucidated, insulin [81]. Infant suckling promotes the secretion of both prolactin and oxytocin. Although cortisol has long been considered essential for mammary differentiation [82], its mechanism of action is poorly understood. While recent work has been carried out in mice and earlier work stemmed from research on animals including goats, sheep, cows, rats, rabbits and sows [83, 84], it is unclear to what extent the mechanisms defined in these studies apply to women. Further, the influence of substrate availability on milk secretion and composition [85] as well as the effects of lactation on systemic metabolism have been widely documented in cows [86], although few studies of the interactions between lactation and systemic metabolism exist in other species. In this section we focus on several issues that are likely to be increasingly important as we seek to produce sufficient human milk of appropriate composition for complete sustenance of infants for 4 to 6 months and partial sustenance thereafter.

- How do hormones and metabolism coordinately program the mammary gland for lactation? (Nommsen-Rivers, Neville, Hinde)
**Research Target: A mechanistic understanding of the factors and signaling pathways that regulate mammary differentiation in late pregnancy, at parturition and during lactation.**

A number of irreversible switches govern mammary gland development: The first is the increase in circulating estrogen at puberty that switches on ductal growth. The hormones of pregnancy, progesterone and prolactin, switch on alveolar development. At mid-pregnancy another switch (secretory differentiation, previously called lactogenesis I) leads to a decrease in proliferation and brings about differentiation of mammary epithelial cells such that they can produce milk during the subsequent lactation. At parturition a fall in progesterone switches on milk secretion (secretory activation, previously called lactogenesis II). Cessation of milk removal at weaning brings about the last switch that induces glandular involution. We have little understanding of the mechanisms by which epigenetic modifications in signaling pathways and gene expression might activate and regulate these switches [87].

The roles of estrogen, progesterone, EGF and related ligands, and prolactin, the receptors for these ligands, and their downstream signaling molecules appear to be well understood [80]. However, modern investigations of regulatory mechanisms in vivo during late pregnancy, at parturition and during lactation, mostly in mice, have been hampered by the problem that the loss of specific growth factors or signaling systems results in an early block of mammary gland development with a total loss of secretory activation and lactation. Further, we do not understand the relevance and application of these mechanistic data derived largely from studies in mice.

While we know how prolactin affects the synthesis of many milk components [88], the signaling pathways and downstream targets for insulin, glucocorticoids and progesterone are less well understood. These hormones are active either in late pregnancy or in lactation, when systemic metabolic factors may also influence their activity. Similarly while the effects of estrogen on ductal development are reasonably well understood, many environmental agents have estrogenic activity, meaning it is important to understand precisely how estrogens modify mammary gland development and lactational competency. We summarize the roles of insulin, glucocorticoids and progesterone here and discuss estrogens in the context of their environmental presence.

### Insulin

Insulin has long been used in culture models of mammary differentiation [82], although the high concentrations likely acted through the IGF1 receptor. Strong evidence now emphasizes that insulin signals through the insulin receptor during lactation [81, 89]. If this is also true of the mammary gland during pregnancy, then the effects of insulin upon epithelial differentiation could substantially influence breastfeeding success. Hypoinsulinemia, as can occur temporarily in Type I diabetes, might interfere with differentiation. The hyperinsulinemia that occurs during obesity and gestational diabetes could hyperstimulate insulin signaling perhaps promoting premature initiation of lactation with subsequent premature involution, since secreted milk could not be removed from the gland. Overexpression of activated Akt, a downstream mediator of insulin signaling had exactly this effect in the mouse mammary gland [90]. Animal studies, possibly in genetically altered mice or larger species more similar to humans such as pigs, along with human studies correlating insulin levels in pregnancy with lactation performance will be necessary to test this hypothesis.

### Glucocorticoids

Although glucocorticoids have long been considered important for milk synthesis [82], they are also associated with the suppression of milk synthesis and milk letdown under stressful situations [91]. The complex effects of glucocorticoid action on lactation are illustrated by the observation that betamethasone, a glucorticoid, facilitates milk production by mothers of preterm infants if given antenatally within 2 days of birth, but inhibits milk production when given 3 to 9 days prepartum [92]. In a mouse model of deficient glucocorticoid secretion, both pup growth and the expression of milk proteins were compromised [93] highlighting a clear requirement for corticoids for proper lactation.

An important question is the role of acute or chronic stress on glucocorticoid signaling. Non-human primate models will be valuable for future research, given their translational potential for humans. Rhesus macaques produce singletons, like humans, and show substantial individual variation in milk cortisol concentrations [94]. Marmosets (*Callithrix jacchus*), a smaller neotropical monkey more distantly related to humans, are emerging as a valuable model of obesity and reproduction [95] and could be particularly useful for understanding metabolic disease and lactational performance.

### Progesterone

While the proliferative effects of progesterone on alveolar development in early pregnancy have received considerable attention [96] the mechanism by which progesterone represses secretory activation in late pregnancy has been difficult to study because of the paucity of in vitro model systems for mammary differentiation and the complexity of progesterone and prolactin receptor interactions [97]. New in vitro model systems in which mammary differentiation can be studied [96, 98], coupled with studies of animals with mutated receptors or downstream effectors are an important next step in elucidating this aspect of progesterone action.

b. What are the genetic and epigenetic elements that affect heritability of mammary gland development and lactation-related traits? (Contributors: Hadsell, Rijnkels)

- **Research Target: Identify the genetic and epigenetic basis for heritable maternal traits related to lactation success.**

Two modes of inherited traits are now recognized: (1) Traits based on DNA sequence are passed genetically to the offspring. (2) Epigenetic alterations in DNA including DNA methylation or other mechanisms can also be passed to the offspring [7]. The relevance of each for human lactation is discussed below.

### Genetic Traits

Mammary development and lactation reflect a collection of quantitative traits that have a continuously distributed variation. It has been clear for nearly a century that variation in these traits cannot be fully understood without studying both genetic and environmental factors [99]. Studies in dairy animals show that genetics can account for as much as 40 % of the variation in lactation-related traits [100]. Litter weight gain in mice, which is an indicator of lactation success, is also heritable with genetics accounting for as much as 33 % of the observed variation [101]. In addition, milk production by dairy animals and rodents can be increased through genetic selection [102, 103]. In parallel, natural genetic variants in humans that alter mammary gland development or produce inadequate lactation are known. For example, isolated prolactin deficiency in women is an autosomal recessive trait known to produce a lactation defect [104]. Finlay-Marks syndrome, thought to involve mutations in the *Lef1* gene, is an inherited condition that results in the absence of pregnancy-dependent breast development [105]. Mutations in the gene SLC30A2 that specifies a zinc transporter in the mammary gland have been documented to result in milk with low zinc concentrations [106, 107]. A genetic variation in FADS2 leading to altered fatty acid profiles in human milk was discussed in Topic II [66]. Despite these examples, our understanding of the role of natural genetic variation in determining mammary gland development and lactation in humans and species other than dairy animals is very limited. Work in mice has led to the identification of lactation quantitative trait loci [108] although the specific genomic loci have yet to be established. Lactation success in humans varies by race or ethnicity [109] although again the specific genetic differences are not known.

In the 11 years since the publication of the human genome enormous advances have been made in cataloging the variation that exists both in humans [110] and in animal models such as the mouse [111, 112]. These resources have already been applied to other aspects of human health and disease. A concerted effort is needed to employ these genomic resources toward understanding mammary gland development and lactation in humans and animal models.

### Epigenetic Changes

An interesting question is whether programming of mammary cells occurs in utero or during lactation in response to maternal nutritional status or other maternal factors. This programming might potentially affect the development and/or lactation performance of the offspring resulting in epigenetic inheritance of lactation traits. Maternal nutrition during pregnancy and lactation can affect the susceptibility of female offspring to mammary tumorigenesis in animals [113, 114]. The nutritional status of gestating sheep produced heritable effects on lactation success in two generations of female offspring [115, 116]. Are such inherited epigenetic changes in mammary development a cause of lactation difficulties in obese women? The answer to this question is particularly important as we seek to reduce the consequences of the obesity epidemic.

c. What are the mechanisms by which maternal nutrition, disease, and metabolic status affect milk composition and volume? (Contributors: Anderson, Friedman, MacLean, McManaman, Van Houten)

- **Research Target: Carefully controlled studies in humans and relevant animal models to dissect the effects of nutrition, maternal adiposity, and metabolic disease on lactation performance.**

Maternal obesity in humans increases the risk of unsuccessful initiation of breastfeeding and impaired ability to sustain lactation once initiated [117, 118]. A similar lactation impairment with obesity was observed in mice fed a “Western” diet, where obese dams produced lipid-poor milk that attenuated litter growth, compared to litters of lean dams on this same diet [9]. As an endocrine disorder obesity has the potential to impact breastfeeding and lactation at several levels including impaired hormonal regulation of mammary gland development [119], altered synthetic and secretory function [9, 120] and disrupted neonatal appetite control mechanisms [121], all impacting higher order integration of infant feeding needs with milk production and ejection [119]. We also need to understand whether the mammary adipose tissue produces locally effective concentrations of estrogen in obese women that could alter mammary gland development and lactation.

Animal models such as rodents (for gene targeting studies) and pigs (for nutrition and endocrine studies) as well as new quantitative approaches in humans that take the mother-child unit into account as an integrated physiological unit need to be developed. The opportunity also exists to incorporate findings from several decades of research on dairy animals into these investigations.

d. What breastfeeding practices prevent inadequate lactation performance? (Contributors: Morrow, Nommsen-Rivers, Meier, Bunik)

- **Research Target: A fundamental understanding of the biological and psychosocial causes of inadequate lactation performance including delayed onset of lactation and consistent low milk production. Also the effect of low milk production during weaning on the composition of milk.**

Inadequate lactation performance may reflect deficiencies in mammary gland development and initiation of lactation as well as ineffective milk removal from the breast by the infant. The mechanisms underlying delayed onset of lactation, lactation failure, and low milk production are poorly defined in part because such deficiencies are rarely investigated due to the ready availability of formula.

Mothers of preterm infants [92], obese women, and women with diabetes and other diseases find initiating and sustaining milk production to be difficult [117]. It is known that stress during labor delays the initiation of lactation [122] and epidural anesthesia during labor interferes with milk letdown [123]. As stated earlier, cortisol given during impending preterm labor affects milk production in variable ways depending on the time elapsed between injection and birth of the infant [92]. Do these observations reflect changes in the initiation of lactation or the ability to produce adequate milk having an appropriate composition? Few studies have been designed to differentiate between these two alternatives, which undoubtedly have different mechanistic bases.

Research on the role of maternal obesity and the effects of stress in lactation performance are priority areas. In both cases it is likely that an altered maternal endocrine environment and possibly proinflammatory cytokines are at least partially responsible for lactation impairment. In the case of obesity the roles for insulin and prolactin [124] require detailed investigation. A pumping regimen for breast milk expression [125] should be tested in prospective studies to determine whether a regimen like that used for mothers of preterm infants can improve milk production. Along with studies of such an intervention, ultrasound or MRI measurements of active breast tissue coupled with plasma levels of relevant hormones and proinflammatory cytokines would lay the groundwork for understanding the biological basis of these problems.

### Milk Production in the Presence of Complementary Feeding

A question which often arises is “what is the effect of complementary foods on milk production and composition”. In a 1991 study [25] milk production fell as mothers decreased the number of daily feeds and milk volume was less than 100 ml per day when daily feeds were fewer than two per day. More importantly, at milk volumes below 200 ml per day, significant increases in the concentrations of protein, sodium and potassium and a decrease in lactose concentration were observed. An important question is whether the protein and carbohydrate composition in the presence of low milk volumes is altered in such a way that the concentration of protective substances like lactoferrin, lysozyme, IgA, and oligosaccharides is increased. If so, continuation of breastfeeding as long as possible after introduction of complementary foods may be important for populations in developing countries.

### The Role of the Infant in Milk Production

Milk removal has long been known to be a major factor in determining the amount of milk secreted by both humans and animals [80]. Infants with ineffective and/or inefficient sucking patterns do not provide the physical breast stimulation and milk removal needed for continued milk synthesis. Thus, ineffective suck can be a key element in lactation failure in both humans [126] and animals [127].

Infant suck is adversely affected by infant morbidity and prematurity [128]. Late preterm infants are at particularly high risk [129] since these infants are often cared for in the general maternity setting where breastfeeding is managed according to guidelines for healthy term infants. Maternal medication and drug use [130] and infant ankyloglossia [131] are also believed to contribute to ineffective infant suck. A systematic research approach is necessary to define elements of lactation failure related to these problems in the infant.

e. How do environmental agents and pharmaceuticals affect milk volume and composition? (Contributors: Badger, Hovey, Horseman)

- **Research Target: Determine the effects and long-term implications of drugs, hormones or phytochemicals (especially dietary factors) on lactation.**

The developing mammary gland is affected by a variety of environmental factors including xenobiotics such as phytochemicals present in common fruits and vegetables [132, 133]. Semi-purified or processed whey proteins used in infant formulas can have similar effects [134, 135]. Although this research area is relatively new, it is now clear that such substances, whether in medications or in the diet, can act through signaling pathways that regulate mammary development. For example, feeding diets rich in soy or whey proteins can have effects similar to those induced by estrogen [136, 137]. Furthermore, there is widespread use of herbal agents, phytochemicals, off-label pharmaceuticals, and even alcohol by women who have a real or perceived difficulty to lactate, yet the full consequences of these agents are unknown.

Numerous environmental factors likely affect initiation of lactation or milk secretion; most significant among these are pharmaceutical agents taken by the mother. For example, in one study of women initiating breastfeeding the use of SSRI’s (selective serotonin reuptake inhibitors, psychotropic drugs) resulted in a substantial delay in the onset of lactation [138]. No mothers using an SSRI initiated lactation before 72 h postpartum. Drugs can also impair milk ejection, diminish milk volume, and alter milk composition. Animals can be very useful for studying the mechanisms of these effects; for example it has been found that dopaminergic agonists inhibit prolactin release in dairy cows [139] and beagles [140]. Although the effects of many of these compounds on breast cancer have received considerable research attention, studies of their effects on lactation are poorly represented in current research agendas.

## Topic IV: Breastfeeding and the At-Risk Neonate

For the purposes of this review at-risk populations include, but are not limited to, low-birth weight/preterm infants, offspring born to obese women or women with diabetes during pregnancy, and infants born in low-resource settings characterized by chronic disease and pandemic infection, food insecurity and poor sanitation. Because the benefits of human milk are likely to be magnified in these groups and settings, such studies are likely to provide opportunities to improve our understanding of mechanisms involved in the effects of milk constituents on infant health and development. Such studies should also clarify the importance of utilizing human milk or substitutes in these populations as well as the nature of effective substitutes.

- What is the role of human milk in the nutrition of the preterm infant? (Contributors: Meier, Neu).
**Research target: A comprehensive understanding of the components of human milk responsible for reducing the risk of morbidities for preterm infants, the mechanisms by which they act, and how they are affected by common NICU practices for collecting, storing, handling and feeding human milk.**

Many organ systems are immature in preterm infants, particularly those who are born very low birth weight (VLBW; <1,500 g birth weight) and extremely low birth weight (ELBW; <1,000 g birth weight). These infants have substantially diminished stores of micro- and macronutrients that are ordinarily deposited during the last trimester in utero. These stores are rapidly depleted after birth due to the co-existence of morbidities that compound the difficult problems already associated with the nutrition of these very small infants. Immediately after birth, ELBW and VLBW infants receive parenteral nutrition with small volumes of enteral nutrition that are introduced and advanced as tolerated by the infant. However, even if full enteral nutrition is achieved, human milk does not meet the micro- and macronutrient requirements of these infants because of their high nutrient demands and the limited milk volumes they can safely ingest. Thus, commercial fortifiers and other exogenous supplements must be added to human milk to provide additional nutrients for ELBW and VLBW infants.

The fact that adequate early nutrition in the smallest preterm infants is subsequently linked to better neurodevelopmental outcomes, highlights the importance of preventing, recognizing and correcting nutrient deficits soon after birth. However, evidence-based strategies for doing so are limited. Feeding human milk to these infants does reduce the risk of serious and costly neonatal intensive care unit (NICU)-acquired morbidities such as NEC and late onset sepsis. Human milk also promotes intestinal [12], cognitive [141] and immune [142] development. An important mechanistic element may be the development of an appropriate intestinal microbiome [143]. There may also be critical periods during the NICU hospitalization when human milk is most important--delineating these periods remains a priority for research [144] as does the practical matter of collecting, storing, handling and feeding human milk.

As emphasized in section I, while a clear understanding of how milk components exert their effects is crucial, it is also imperative to understand the within- and between- mother variation in these components, and to determine the mechanisms underlying this variation. Potential influences include duration of gestation as well as genetic and dietary factors. Defining the temporal changes in human milk composition is critical for the design of regimens for feeding human milk to the premature infant in the NICU where expressed milk is seldom fed in any particular order [145]. While certain types of studies (for example, defining the microbiota) can be done using preterm infants as an experimental model, for many studies an animal model is required. Here a role for the neonatal pig is assuming greater importance [31, 146].

Because it is often difficult to obtain sufficient quantities of human milk from mothers of preterm infants, we also need to understand how to optimize milk synthesis and removal in these mothers, most of whom are breast pump-dependent for weeks or months. Mothers of preterm infants have documented risk factors for delayed onset of lactation and low milk volume, but the mechanisms underlying these problems as well as their diagnosis and management are poorly understood. Because preterm infants benefit from even small enteral feedings as soon as they are able to tolerate them, donor human milk is frequently recommended. However, many questions remain about the use of donor human milk including which infants should receive it, for how long, and where a source of suitable donors can be created [147].

Finally, a risk-benefit assessment model for the use of therapeutic drugs for maternal diseases is critical. A useful model would allow caregivers to balance the effects of giving human milk vs formula against the potential negative effects of drug therapy essential for maternal health on both milk secretion and infant development.

b. What is the role of breastfeeding in preventing obesity in susceptible populations? (Contributors: Dabelea, Crume, MacLean, Friedman)
**Research target: Understanding the mechanisms by which breastfeeding modifies the fetal programming of infants at risk for metabolic disease as adults.**

Infants with intrauterine growth retardation as well as those born to obese mothers, to mothers with gestational diabetes, and to those who are malnourished are more susceptible to developing the metabolic syndrome later in life, including type II diabetes and heart disease [148, 149]. The extensive literature on fetal programming for metabolic disease will not be reviewed here. The important consideration is the role of breastfeeding in abrogating undesirable fetal programming.

### The Effect of Breastfeeding in the General Pediatric Population

Studies assessing the influence of breastfeeding on the risk of childhood overweight or obesity have demonstrated significant protection [150], but the effect of breastfeeding has been smaller and more variable when the outcome is assessed as a difference in mean BMI. These findings led some researchers to conclude that the effect of breastfeeding is small and influenced by bias and confounding. Importantly, in a recent study [151] school entry data on 14,412 children aged 4.5–7 years in southern Germany were broken down by BMI percentile. After adjusting for a large number of potential confounding variables, mean BMI was significantly reduced in children above the 90th BMI percentile who had been breastfed versus the formula-fed children in this BMI group. Interestingly, among children in the lower BMI percentiles (≥30 %), breastfeeding was associated with a low but significant shift towards a higher BMI. These results suggest that breastfeeding prevents overweight and obesity in children with a tendency to these outcomes, but does not affect underweight children in terms of weight reduction. In a very recent study of growth trajectories for offspring of non-diabetic pregnancies slower growth velocity was observed in those infants who were breastfed [152]. The effects of prior breastfeeding were particularly significant at ages 6 to 13 years. Importantly the population in this study was mostly middle class; extending this type of research to lower socioeconomic groups where obesity tends to be more prevalent is critical.

### The Effect of Breastfeeding in the Children Exposed to Maternal Diabetes and Obesity

The potential of breastfeeding to reduce the risk for obesity following in utero exposure to overnutrition from a diabetic pregnancy or maternal obesity has great public health significance. Among Pima Indian youth exposed to maternal Type 2 Diabetes or gestational diabetes those who were breastfed for at least 2 months had a lower risk for developing diabetes compared to those who were formula- fed (30.1 vs. 43.6 % risk) [153]. In a recent comparison [154] of middle class children exposed to maternal diabetes in utero a significant decrease in childhood BMI and central adiposity was observed among children who were breastfed according current recommendations. A similar (25 %) reduction in childhood obesity risk was associated with exclusive breastfeeding for 6 months compared to exclusive formula feeding [155]. Children who received a mix of formula and human milk had results similar to the formula-fed group. Similar results were reported from 2 to 14 year old children in the National Longitudinal Survey of Youth [156]. *All these results are consistent with the conclusion that children at risk for childhood obesity stemming from exposures* in utero *benefit significantly from breastfeeding.*

### Potential Mechanisms

The increasing number of observations that breastfeeding alters the growth trajectory of offspring born from both diabetic and non-diabetic pregnancies suggests that early infant diet has an important long-term effect [157]. Understanding the mechanism of these effects is important in determining what potential interventions might be effective. The complexity of the issue is revealed by both human and animal studies. For example, a quantitative relationship between consumption of milk from diabetic mothers and the risk of overweight at 1–5 years of age has been observed [157] suggesting an effect of diabetes on milk composition. When neonatal rats born to lean dams were cross-fostered to obese dams they developed an obese phenotype, including insulin resistance [158]. Maternal obesity in mice was shown to alter milk lipid content leading to decreased neonatal energy intake and decreased weight gain [9].

The question becomes What *is the mechanism?* Human milk contains more lactose, specific fats and cholesterol and relatively lower protein and mineral contents compared to milk from other animals. It is also possible that specific micronutrients or hydrolysates of macronutrients present in human but not cow’s milk stimulate GI hormones that act on the hypothalamus to regulate appetite. In support of this notion, babies who were given cow’s milk formula with added glutamate or hydrolyzed formula consumed fewer calories than those who drank regular cow’s-milk formula [159]. The influence of the neonatal microbiome on development of appetite regulation has been hinted at in rodent studies, but the mechanism is poorly understood. An answer to the question of whether satiety hormones such as leptin, adiponectin, and ghrelin or pro/anti-inflammation components of human milk have effects on infant adiposity, appetite, and energy expenditure could go a long way toward defining the early-life risk factors that subsequently lead to adiposity.

In summary, breastfeeding may be a powerful strategy to decrease the risk of obesity among offspring of mothers with pregnancy-associated diabetes or obesity during pregnancy. Additional research is needed to confirm this finding, to define compositional differences in human milk from healthy and diabetic mothers, to assess the influence of combination feeding with formula and to find the mechanism(s) involved. In addition, if breast-feeding has a significant effect on childhood obesity, principally for infants born to obese mothers, it will be important to find ways to increase the rate of breastfeeding in this population, particularly given significant evidence that obese women have problems initiating and maintaining lactation [117].

c. What is the role of human milk in preventing growth stunting? (Contributors: Hambidge, Krebs)
**Research Target: To develop nutritional programs that will prevent/ameliorate growth stunting of malnourished infants.**

Growth stunting is a serious problem in developing countries where it is associated with poor cognitive and educational performance [57]. This condition, if not addressed prior to the third year of life has deleterious effects on height, strength and intellect in adult life, reducing what Vitora has called “human capital” [8]. The problem is that while breastfeeding is associated with improved survival in this population, it does not prevent stunting.

Progress has been made toward increasing the rates of exclusive breastfeeding for approximately the first 6 months of life in low resource settings and is predicted to have a major impact on infant survival (1). However, recent observations have highlighted faltering of very early postnatal linear growth occurring even during the period of exclusive breastfeeding [160]. Contrary to expectations, in most instances the rate of energy intake is not the growth-limiting factor. A possible causal effect on early growth faltering is micronutrient inadequacy in milk from undernourished mothers particularly on the heels of micronutrient deficiencies acquired by the fetus. Other possible explanations include deleterious bioactive compounds in human milk as a result of a pro-inflammatory environment due to maternal metabolic dysregulation. Additional or alternative factors in the intra-uterine or pre-conceptional environment may contribute to altered infant growth patterns [161]. However, all these possibilities are poorly defined.

After approximately the first 4 to 6 months of near exclusive breastfeeding the infant becomes dependent on other sources to meet its requirements for iron and zinc. These requirements are predictable due to depletion of infant iron stores by this time and the physiologic decline in zinc concentrations in human milk, respectively [162]. Broadly speaking, appropriate options are complementary foods, fortified foods, or supplements [163]. Meat provides both iron and zinc in a bioavailable form. Alternatively, fortified cereal can be an acceptable and efficacious vehicle, although the enteric microbiome and inflammatory responses appear to differ from those achieved on meat diets. In sum, research in this area has not yet found solutions to the problem of appropriate complementary foods in the undernourished population [164].

These observations illustrate the complexity underlying perturbed infant growth patterns in disadvantaged populations, with likely interplay among nutritional, immunostimulatory, and metabolic factors. The choice of interventions must take into account infant nutritional status as well as the nutritional and metabolic status of the mother. Better data that improve our ability to make rational nutritional choices for the undernourished infant will have a profound global impact on long term health.

d. A community approach to lactation studies in at-risk human populations. (Contributor: Seewaldt)
**Research Target: Conduct meaningful clinical studies in at-risk populations who may have low socioeconomic status, poor health, poor nutrition and a myriad of problems that prevent consistent access and participation.**

As stated above, studies in at-risk populations are more likely to yield statistically meaningful results about the benefits of breastfeeding. However, these populations are often difficult to access in a consequential way, particularly for studies with a longitudinal component.

One particularly effective approach has been developed for studying young women in a community that as a whole is at high-risk for triple-negative breast cancer [165]. A longitudinal study of breast cancer initiation in this population was made possible by partnering with community advocates and navigators. Key components of the partnership included

1. Support for high-quality free breast cancer screening at community clinics
2. Partnership with breast and cervical cancer control programs
3. Free or low cost follow-up and treatment services
4. Community navigators
5. Mentorship of minority scholars.

Taken together, these components, as well as a willingness to partner and listen, are essential for collaborating to develop community-partnered studies.

Importantly, breastfeeding rates in this underserved population are low and, for those women who do choose to breastfeed, duration is short with many discontinuing within days after birth [166]. Factors that are hypothesized to influence breastfeeding incidence in this population include aggressive marketing of human milk substitutes, workplaces that discourage breastfeeding, social and personal networks and cultural norms, and individual beliefs about breastfeeding [166]. However, in some populations biological deficiencies in mammary development may underlie lack of successful breastfeeding. Effective studies of this problem will require a community partnership like that developed for the study of breast cancer.

### V. Training the Future Lactation Biologist (Contributor: Shur)

- **Educational Target: New programs and resources to repopulate the diminishing pool of lactation biologists and promote communication between the diverse disciplines required to understand milk, its secretion and its effects on the neonate.**

The pool of highly-trained investigators qualified to study problems in lactation biology has been diminished due to a variety of factors. Historically, many land grant colleges had formal departments and training programs in dairy and animal sciences, often funded by the USDA. Because milk production worldwide now exceeds demand and formula is widely available, revenue for studies of lactation biology has decreased dramatically. The result has been the reduction, if not closure, of some of these departments, and a concomitant disappearance of training programs in lactation biology. Further, support from federal funding agencies such as NIH is often targeted toward disease rather than studies of normal biology. In the field of mammary gland biology and lactation the majority of this support is directed toward breast cancer rather than lactation biology. In addition a lack of formal clinical training in lactation biology in medical school curricula coupled with the cancer orientation of conferences and other traditional venues means that there are few places where scientists in all disciplines that now require knowledge of lactation biology can exchange information and ideas. **Therefore, we need to implement new programs and resources to repopulate the diminishing pool of lactation biologists.** In particular, federal funding agencies must make a conscious commitment to set aside resources to rebuild this cadre of investigators through new training programs, workshops, conferences, travel grants, and similar initiatives as described below.

- Compelling reasons to pursue training in lactation biology.

With a better appreciation of the benefits of breastfeeding relative to formula, there has been a renewed interest in the basic biology of lactation. In addition federal funding agencies are now using their limited resources to focus on problems that directly impact human health including disease prevention, for which the basic biology of the mammary gland is a prime example. Furthermore, a fundamental understanding of lactation biology impacts a wide range of disciplines, ranging from development, to biochemistry of lipid and complex carbohydrates, to cell biology, to physiology, to behavior, to clinical medicine. This understanding is well- suited to the widespread new emphasis on multidisciplinary “team” approaches to complex scientific problems. There is also an emerging paradigm shift that animal models no longer should be exclusively limited to rodents. Indeed, the study of lactation is unique in that it is a process common to all mammals, meaning there are certainly unexplored opportunities to address many of the above-mentioned research targets in species besides rodents. This approach will however require future scientists to be trained in a variety of animal models.

b. Skills required of the next generation of investigators.

In this climate of limited resources, predoctoral training programs should focus on the basic biomedical foundations relevant to studies of lactation biology, rather than exist as specialized training programs in this multifaceted discipline. Predoctoral students interested in lactation biology should receive training in endocrinology and integrative physiology, nutrition, biochemistry, cell biology, molecular biology, innate immunity, complex carbohydrates, bioinformatics, genetics, and other relevant disciplines. For broader application to breastfeeding it may be useful for students to be trained in research approaches to human development, behavioral psychology, sociology, public health and epidemiology.

c. Training the next generation of investigators.

#### Postdoctoral Training Programs

Unlike predoctoral training programs, postdoctoral training programs are an ideal venue for specialized training in lactation biology, at which time trainees should be exposed to the non-biomedical, psychosocial aspects of lactation. Examples include the impact of maternal behavior and stress on the quality of lactation, as well as issues pertaining to health disparities that impact the frequency and quality of lactation. In this regard, federal agencies should set aside resources to provide training for postdoctoral fellows, via both individual and institutional training programs. Federal agencies should encourage cross-institutional and cross disciplinary collaborations that bring together experts representing the broad range of disciplines and species relevant to studies in lactation biology. These groups should include both basic and clinical investigators and provide interactions with experts in the psychosocial aspects of breastfeeding and the problems of providing adequate nutrition for at-risk infants.

#### Focused Courses and Workshops

Funds need to be made available for “hands-on” workshops in order for postdoctoral fellows and new investigators to receive state-of-the-art training. An intensive hands-on program could be modeled after those offered at Woods Hole in Physiology, among other disciplines. Such workshops could include basic techniques in lactation biology, overviews of clinical lactation topics, comparative model systems, compositional analysis and, bioinformatics. Ideally such a meeting might best take place at a site that could offer complementary perspectives from both dairy science and medical science. In this regard, funds need to be made available to support the travel and registration for trainees and new investigators in order to participate in these programs.

#### Venues for Sharing Ideas/Resources

Trainees interested in lactation biology need a “safe haven” to share ideas, their work, problems, etc. Many of the current conferences are dominated by senior investigators, and consequently, a “pre-conference” workshop for new investigators, such as is currently done at the Gordon Research Conference, ISRHML and the meetings of the International Milk Genomics Consortium would provide an opportunity to build community and collaborations. Similarly, the professional societies should develop trainee divisions, similar to that at the American Dairy Science Association to serve the needs of new trainees and investigators. One issue faced by the existing meetings is that they generally address the needs of only a single component/perspective of the lactation biology community. After meeting in Denver to develop this white paper, it was clear that there is a need for occasional but regularly-scheduled, cross-disciplinary meetings that embrace the diversity of perspectives and thoughts enjoyed at this conference. Finally, a simple online “newsletter” or “blog” could be developed as a “real-time” means to share resources, ideas, reagents, and/or problems among new and established investigators alike.

## Section VI. Imperatives for Advancing the Field

There is a tremendous need for additional evidence that will allow the clinical management of lactation and breastfeeding support to be better tailored to the needs of the maternal-infant dyad. Milk, especially human milk, is increasingly revealed as a complex substance that exerts effects far beyond its nutritional value. Studies of the mechanisms by which milk components affect all aspects of infant development are themselves in their infancy. The psychosocial factors that affect a mother’s ability to breastfeed and the psychological effects of the act of breastfeeding upon both mother and infant are poorly defined. While these problems are complex, three principles emerged from the far-ranging discussions at this conference:

1. There is an urgent need to define the precise function of the various components of human and other milks as well as their interactions.

Aside from a small number of components such as lactoferrin, the inter- and intra-individual variation in milk composition as well as the mechanisms of action of many bioactive components of human milk remain poorly characterized. Studies of these topics are crucial for understanding how human milk facilitates resistance to infection, establishes the gut microbiota, develops the immune system and facilitates cognitive development.

Principle 2: The role of breastfeeding in infant nutritional status must be studied in light of the changing dynamic between mother and child from preconception through pregnancy and into postnatal life.

It is important to recognize the intimate and integral role of the maternal-infant biological and social unit throughout pregnancy and through lactation and the period of complementary feeding.

Principle 3: Priority should be given to studying the impact of breastfeeding in high-risk populations.

The imperatives listed in Table 1 were deemed to be consistent with these principles and of most importance for future research.

**Table 1:** Imperatives for future milk and lactation research

	Topic	Models/Priority Targets	Goals
1	Milk Biochemistry	Humans, dairy species	*Describe the structure of all human milk components as well as their variability within- and among women; determine composition of bovine milk as basis for substitutes.*
2	Functional Properties of Milk Bioactives		
	Intestinal Development	Humans, pig, tissue culture	*Determine the bioactive components of milk and the mechanisms of their effects on GI development, microbiome and GALT as well as their anti-infectious activities*
	Immune Tolerance	Human	*Identify critical windows for development of immune tolerance or inflammatory reactivity to allergens as mediated by early life nutrition*
	Cognitive Development	Human, monkey, rodent, pig	*Investigate cognitive and behavioral development as a function of milk constituents.*
	Stunting/Impaired Growth	Undernourished infants	*Examine role of human milk, combined with maternal and infant interventions, for counteracting growth stunting in developing countries and elsewhere*
3	Lactation	Human, rodent, monkey, livestock, tissue culture models	*Determine molecular and cellular mechanisms by which genetic, physiologic, environmental, behavioral, and societal factors affect mammary development, milk production and composition with emphasis on endocrine agents and metabolic factors.*
4	Very low birth weight infants (VLBW)	Pre-term, neonatal infants, pig	*Identify the components of human milk responsible for reducing the risk of morbidities in preterm infants, the mechanisms by which they act, and the efficacy of common practices for collecting, storing, handling and feeding human milk for this population*
5	Maternal Obesity	Human, pig, rodent	*Understand the mechanism by which obesity affects mammary gland development, milk secretion and milk composition; determine how breastfeeding affects obesity programming in offspring.*
6	Breastfeeding Practices		
	Initiation	Humans, particularly at-risk populations	*Utilize quantitative methods to assess effects of obstacles to breastfeeding on initiation of lactation and duration of breastfeeding in low income women and women with co-existing health problems. Determine the impact of exclusive breastfeeding beyond 12 weeks on mother and infant*
	Duration		
	Exclusivity		
7	Valuation	Humans	*Assess economic impacts* via *direct and indirect costs*
8	Scientific Interactions/Training	Courses, meetings	*Support ongoing forums for discussion among students, researchers, and faculty; NIH training and conference grants; increase interactions between researchers in the human lactation and dairy fields.*

## Abbreviations

AA: Arachidonic acid
DHA: Docosahexaenoic acid
ELBW: Extremely low birth weight, <1,000 g birth weight
GI: Gastrointestinal
HFD: High fat diet
HMO: Human milk oligosaccharides
IL-10: Interleukin-10
LC-PUFA: Long chain poly unsaturated fatty acids
NEC: Necrotizing enterocolitis
NICU: Neonatal intensive care unit
sIgA: Secretory immunoglobulin A
TGF: Transforming growth factor
VLBW: Very low birth weight, <1,500 g birth weight
